# Therapeutic Potential of Kappa Opioid Agonists

**DOI:** 10.3390/ph12020095

**Published:** 2019-06-20

**Authors:** Tyler C. Beck, Matthew A. Hapstack, Kyle R. Beck, Thomas A. Dix

**Affiliations:** 1 Drug Discovery & Biomedical Sciences, Medical University of South Carolina, 280 Calhoun Street, QF204, Charleston, SC 29424-2303, USA; beckt@musc.edu; 2 College of Medicine, 173 Ashley Ave., Charleston, SC 29424-2303, USA; hapstack@musc.edu; 3 College of Pharmacy, The Ohio State University, 500 W 12th Ave, Columbus, OH 43210-9998, USA; beck.735@osu.edu; 4 JT Pharmaceuticals, Inc., 300 West Coleman Blvd., Suite 203, Mount Pleasant, SC 29464-2303, USA

**Keywords:** therapeutic, potential, indications

## Abstract

Many original research articles have been published that describe findings and outline areas for the development of kappa-opioid agonists (KOAs) as novel drugs; however, a single review article that summarizes the broad potential for KOAs in drug development does not exist. It is well-established that KOAs demonstrate efficacy in pain attenuation; however, KOAs also have proven to be beneficial in treating a variety of novel but often overlapping conditions including cardiovascular disease, pruritus, nausea, inflammatory diseases, spinal anesthesia, stroke, hypoxic pulmonary hypertension, multiple sclerosis, addiction, and post-traumatic cartilage degeneration. This article summarizes key findings of KOAs and discusses the untapped therapeutic potential of KOAs in the treatment of many human diseases.

## 1. Introduction

Opioid analgesics have been used for thousands of years in the treatment of pain and related disorders, and have become among the most widely prescribed medications in use today [1]. In 2016, there were an estimated 67 prescriptions per 100 citizens in the United States. The total opioids, in morphine milligram equivalents (MMEs), prescribed per year has nearly tripled since 1999 [2,3]. The analgesic effects of opioids are mediated through four known receptors: mu-(MOR), kappa-(KOR), delta-(DOR) and opioid receptor-like 1 (ORL-1); all four are members of the seven transmembrane spanning G-protein coupled receptor family [1]. Of the opioid analgesics, mu-opioid agonists (MOAs) are the most commonly used and are indicated for a variety of conditions, including acute and chronic pain management, cough, and diarrhea. However, MOA use results in a plethora of well-described side-effects; these include nausea, vomiting, constipation, respiratory depression, addiction, tolerance, and sedation. Promising alternatives to MOAs are kappa-opioid agonists (KOAs); these agents have indistinguishable analgesic properties and a reduced side-effect profile. Development of novel KOAs has been limited due to untoward side-effects mediated by centrally-located KORs, including dysphoria, sedation, and hallucinations. However, efforts to develop novel KOAs that preferentially target beneficial signaling pathways have provided a way to mitigate the unwanted side-effect profile associated with conventional KOAs, such as salvinorin [4]. Additionally, recent efforts to develop peripherally-restricted KOAs with limited central nervous system (CNS) penetration have provided means to reduce the adverse effects and toxicities of typical opioids. Both approaches offer immense potential for the development of novel opioid agonists fit for numerous indications.

**Figure 1:**
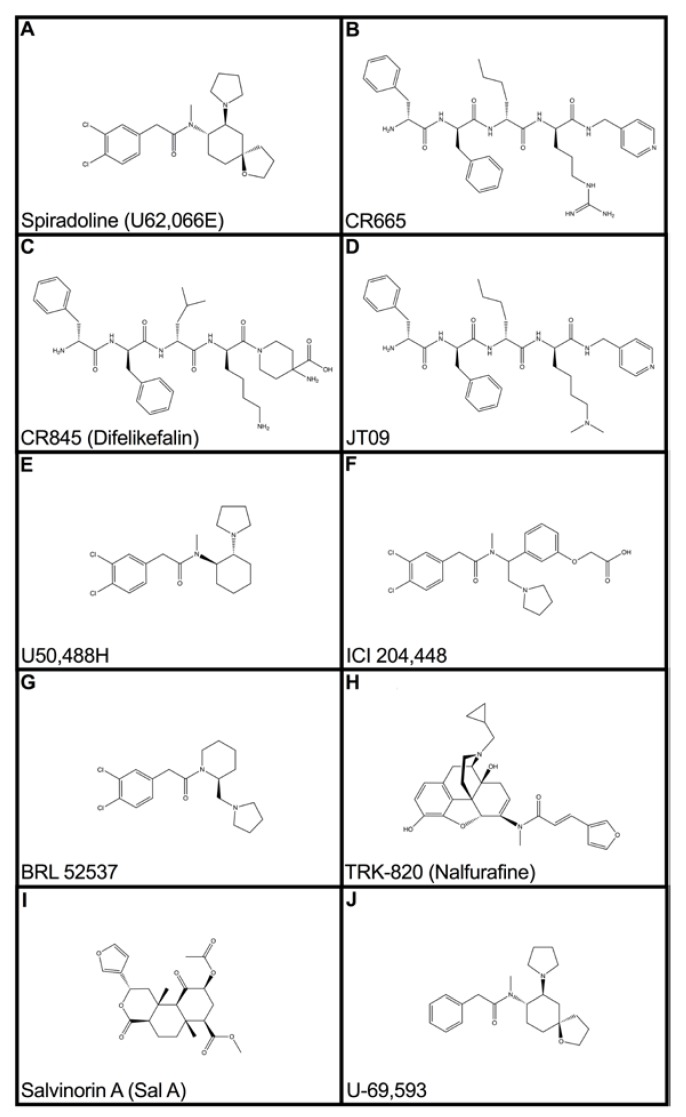
Structures of well-described kappa-opioid receptor agonists and related compounds. The compounds CR665 (**B**), CR845 (**C**), JT09 (**D**), and ICI 204,448 (**F**) are peripherally-selective kappa-opioid agonists (KOAs). Spiradoline (**A**), U50,488H (**E**), BRL 52537 (**G**), TRK-820 (**H**), Salvinorin A (**I**), and U-69,593 (**J**) are centrally-active KOAs.

The opioid receptor signaling pathways have been well understood for the past few decades. The opioid receptor system activates inhibitory G-proteins, forming homo- and hetero-dimer complexes, which signals downstream kinase cascades in order to scaffold a variety of proteins [1]. However, we currently lack understanding related to the diversity of signaling at opioid receptors, such as KOR, and how exactly the second messengers act to modulate pain and behavioral activity. Furthermore, there have been several supplementary mechanisms of action proposed that are discussed in this review. Developing a deeper understanding of these pathways could provide future direction in the discovery of pathway-specific novel drug entities. Several KOAs exist (Figure 1) and have been studied extensively in animal models of pain; however, research today continues to reveal novel actions of these compounds. The primary aim of this article is to promote a heightened understanding within the scientific community related to the significance of the KOR and to define future avenues of development related to such compounds.

## 2. Indications

### 2.1. Chronic Pain

Chronic pain is the most common reason patients seek medical care, and it costs society an estimated $635 billion dollars annually [5]. It is most frequently managed with MOAs, which are associated with many side-effects, most notably, addiction. On average, 115 people die every day from an opioid overdose [6]. Thus, the need for a non-addictive analgesic is clear. KOAs have been cited as the most efficacious of the opioids in attenuating visceral pain, yet their development has been widely discontinued due to the promotion of untoward side-effects [7]. Evaluation of early non-peptidic KOAs resulted in centrally-mediated sedation and dysphoria [8]. Of note, KOAs and MOAs have demonstrated a synergistic antinociceptive effect in rat models of visceral pain [9]. Rech et al. at Michigan State University demonstrated that co-administration of the MOR agonist fentanyl and the KOR agonist U62,066E (spiradoline) (Figure 1A) promoted enhanced analgesic activity and a reduced side-effect profile relative to monotherapy treatment with fentanyl [9,10]. A promising alternative is peptide-derived KOAs, including CR665 (Figure 1B), CR845 (Difelikefalin^®^) (Figure 1C), and JT09 (Figure 1D), which exhibit peripheral selectivity and have shown benefits in patients with visceral and neuropathic pain [7,11,12]. These drugs have the same analgesic effects as the early KOAs without the negative side-effects mediated by central receptors. CR665 and CR845, under development by Cara Therapeutics (Stamford, CT, USA), are potent and long-acting peripherally-selective KOAs; however, they lack sufficient oral bio-availability [7,11]. In pre-clinical studies, CR845 alleviated hyperalgesia in a rat model of inflammatory pain; it also inhibited tactile hypersensitivity in a rat model of neuropathic pain [9]. CR845 is currently in phase III clinical trials for the intravenous management of acute post-operative pain and uremic pruritus. CR845 has demonstrated an oral bio-availability of 15%, thus, Cara Therapeutics currently is attempting to reformulate the drug to enhance its gastrointestinal stability and absorption. JT09, being developed by our research group at the Medical University of South Carolina and JT Pharma (Mount Pleasant, SC, USA), is a second-generation compound to CR665 [7,12]. JT09 is a potent, long-acting, peripherally-selective KOA with significant oral bioavailability. Both CR665 and JT09 feature different modifications of the parent KOA tetrapeptide fragment, but the overall rationalization for the changes are quite similar. For both, substitution of non-natural analogs of the amino acids arginine and lysine leads to less deactivation through cleavage by serum proteases, while also minimizing CNS penetration. The compounds are both selective KOAs and were identified through their activities in rat functional models versus a computer-aided design approach. In pre-clinical studies, JT09 was as efficacious as morphine in alleviating peripheral pain when administered orally to rats (ED_50_: 4.7 mg/kg) without inducing side-effects associated with CNS-penetration (i.e., abuse liability, dysphoria, sedation, etc.) that limit the conventional opioid analgesics [7]. These novel analgesics have the potential to help address the current opioid epidemic.

### 2.2. Myocardial Infarction

Every 40 s, someone in the United States suffers from a myocardial infarction (MI) [13]. Approximately 790,000 Americans have a heart attack annually, costing society $75 billion dollars in direct medical costs associated with acute and chronic medical management [14]. The current standard of care for initial treatment of MI is directed towards rapid restoration of perfusion in an attempt to salvage functional myocardium [15]. Most commonly, this is achieved through percutaneous coronary intervention (PCI) or coronary artery bypass graft (CABG) surgery. Despite these interventions, the 24-h mortality rate of an acute MI is approximately 33%; of survivors, 15% will experience reinfarction within 30 days (10). Over time, approximately 30% of patients will develop heart failure [16].

KOA treatment has demonstrated the ability of these compounds to induce cardio-protection in MI patients. Specifically, they reduce infarct size and decrease the incidence of heart failure following myocardial infarction. Immunostaining for the opioid receptors following sudden cardiac death has demonstrated that the KORs and DORs are upregulated in cardiac myocytes following a cardiac event [17]. It is suggested that enkephalins, which stimulate DORs, and dynorphins, which stimulate KORs, play a vital role in the functional recovery of cardiac myocytes by limiting apoptotic cell death [18]. Exogenous opioid receptor agonists, when applied before, during, or shortly after MI, ameliorate myocardial damage by stimulating G_i_-linked pathways that alter the myocardial ion channel activity and the intracellular activity of protein kinases. Among these methods is ischemic preconditioning (IPC), which involves several brief intervals of coronary artery occlusion and reperfusion. This leads to myocardial ischemia and ATP-depletion, promoting an adaptive response by the myocardium and making it less susceptible to future ischemic insult. IPC is attenuated by opioid receptor antagonists, suggesting that the ORs play a role in IPC. Investigators at the Medical University of Wisconsin evaluated the effects of known selective KOAs on IPC induction. The experimental compounds U50,488H (Figure 1E), ICI 204,448 (Figure 1F), and BRL 52537 (Figure 1G) were administered prior to the in vivo induction of myocardial infarction in rats [19]. Following this procedure, rats were sacrificed and the infarct size was examined by negative staining. Results indicated that all three KOAs significantly reduced infarct size to levels comparable to BW373U86, a known delta-opioid agonist (DOA) that promotes IPC and subsequent infarct size reduction. Additionally, infarct-sparing properties of all three KOAs were abolished by co-administration of nor-binaltorphimine (nor-BNI), a selective kappa-opioid receptor antagonist. This validates the observation that KORs mediate infarct-sparing effects of IPC. These data suggest a potential prophylactic benefit of KOAs in patients that are at risk for MI.

A study conducted by the Department of Cardiovascular Surgery at Guangzhou General Hospital examined the effects of U50,488H on the development of heart failure following myocardial ischemia and reperfusion. In doing so, they subjected male Sprague–Dawley rats to 30 min of myocardial ischemia via left anterior descending artery (LAD) occlusion, followed by four weeks of reperfusion [20]. In comparison to the control group, rats treated with U-50,488H at the initiation of reperfusion had smaller areas of infarcted myocardium, less fibrosis and hypertrophy, reduced oxidative stress, improved mechanical function, and better neovascularization. Using RT-PCR and Western Blot analysis, it was determined that these effects are mediated by the activation of heme oxygenase-1 expression through the PI3K-Akt-Nrf2 pathway. Additionally, it has been demonstrated that the TLR4/NF-κB signaling pathway is activated during myocardial ischemia and reperfusion. This pathway induces upregulation of TNF-α, which promotes a deleterious inflammatory response via cytokine release and neutrophil activation [21]. KOAs, such as U50,488H, have demonstrated the capacity to downregulate the expression of TLR4 and NF-κB, causing a reduction in myeloperoxidase (MPO) and TNF-α levels. As such, KOA-administered rodents experienced decreased myocardial apoptosis and subsequently, a reduction in infarct size. These effects were abolished by nor-BNI, the previously mentioned kappa-opioid receptor antagonist. A study conducted at the University of Hong Kong demonstrated that at low doses, U50,488H had anti-arrhythmic effects, whereas high doses had pro-arrhythmic effects [22]. The infarct sparing effects of U50,488H were observed at low dose levels, suggesting potential benefit of KOAs in attenuating both arrhythmogenesis and myocyte cell death at target dose levels. Lastly, the highly-selective CNS penetrating KOA Spiradoline caused a reduction in heart rate and contractility when administered to rodents, while also increasing the PR interval and QRS width [23]. However, Spiradoline is not used clinically due to the onset of CNS-mediated side-effects associated with KOR agonism, as previously described. Thus, it is imperative that KOAs developed for the indication of cardiovascular disease demonstrate peripherally-selectivity.

KOAs have the potential to be used in combination with other cardioprotective agents. Furthermore, *β*-adrenergic receptor antagonists (*β*-blockers) are used immediately post-MI to decrease the oxygen demand of the myocardium by reducing heart rate, blood pressure, and contractility [24]. At low doses, KOAs administered acutely following myocardial infarction may supplement the inotropic and chronotropic effects of *β*-blockers, while reducing oxidative stress and deleterious inflammation. KOAs may be used chronically with other cardioprotective agents, such as angiotensin II receptor A inhibitors (ACE inhibitors) to attenuate cardiac remodeling and arrhythmogenesis, while enhancing cardiac performance. Additionally, KOAs act as aquaretic agents in promoting diuresis. While diuresis may be seen as an undesired side-effect for some, it may prove to be beneficial in patients who have experienced MI or are in heart failure. The ability of KOAs to promote diuresis could help ameliorate fluid retention and serve to decrease cardiac preload, thus reducing pulmonary and systemic congestion. Thus, we conclude that development of therapeutic KOAs could potentially reduce infarct size following MI by promoting IPC and mitigating ischemia-reperfusion injury, while also reducing the incidence and side-effects of heart failure.

### 2.3. Anti-Pruritic

Pruritus, an irritating sensation promoting the urge to itch, is one of the most common reasons patients seek dermatologic intervention and is thought to be mediated by several mechanisms. Most conclude that pruritus is mediated by pruritogens, which act by stimulating itch-specific C-fibers [25]. Subsequently, these nerve fibers transmit information to the somatosensory cortex, allowing the brain to interpret the itch sensation and provoke a response. Interestingly, opioid receptors have been implicated in the central and peripheral induction of itch. It has been proposed that pruritus may also be mediated by an imbalance of MORs and KORs in central and peripheral pathways. Agonism of MORs promotes the transmission of itch signals to the brain, whereas agonism of KORs inhibits the transmission of itch signals [26]. Researchers at the University of Basel demonstrated an increase in the ratio of MORs to KORs in the epidermis of patients afflicted by chronic pruritus. Thus, administration of either MOR antagonists or KOAs have demonstrated the capacity to attenuate itch sensation. Moreover, the intravenously available KOAs TRK-820 (Nalfurafine^®^) (Figure 1H) and CR845 have demonstrated efficacy in treatment of uremic pruritus [25]. Currently, TRK-820 is approved in Japan for the treatment of uremic pruritus in patients with chronic kidney disease undergoing hemodialysis. CR845 is currently in phase III clinical trials in the United States for the same indication. Additionally, KOAs, such as Spiradoline, demonstrate anti-histaminergic effects and thus, may act synergistically when co-administered with antihistamines, such as diphenhydramine [23].

### 2.4. Anti-Inflammatory and Anti-Edema

Inflammation is a multilevel response to both exogenous infectious agents and endogenous danger signals, such as damage-associated molecular patterns (DAMPs). The inflammatory process is implicated in tissue injury, such as degenerative arthritis, as well as in various auto-immune disorders, including systemic lupus erythematosus, rheumatoid arthritis, Chron’s disease, ulcerative colitis, and many others [27]. Such conditions are incredibly painful and progressively debilitating. The result for many patients is a diminished quality of life and substantial economic impacts. The inflammatory process begins with acute inflammation, which is the immediate response to injury from trauma, infection, or stress. This process helps to prevent further injury and allows for initiation of the healing process. However, if inflammation becomes chronic, medical intervention is necessary in order to prevent permanent damage and continued inflammatory pain. Current treatment for chronic inflammation includes non-steroidal anti-inflammatory drugs (NSAIDs), corticosteroids, and other immunosuppressing agents, all of which have variable efficacy and deleterious side-effects associated with long-term administration. A viable alternative to such agents are KOAs, which have demonstrated the ability to dramatically decrease inflammation and reduce disease severity by as much as 80% [28].

The anti-inflammatory effects of KOAs are exerted by a variety of mechanisms. KOAs reduce cell adhesion molecule expression, inhibit cell trafficking, alter mRNA expression, and reduce levels of substance P (SP) and calcitonin gene-related peptide (CGRP) in joint tissue [28]. The neuropeptides SP and CGRP are seen in the joint tissue during the late phases of arthritis and are thought to be responsible for maintenance of the disease. Further, KOAs reduce the expression of various cytokines, including TNF-α, most likely by inhibiting the TLR4/NF-κB signaling pathway. Other cytokines and cytokine receptors that KOAs have demonstrated the capacity to reduce include IL-1β, IL-2, IL-2 receptor α chain (CD25), IL-6, IL-7 receptor α chain, and IL-10, all of which are implicated in the inflammatory process [29,30]. Additionally, U50,488H-treated cell cultures demonstrated significant elevation in expression of chemokine receptor CCR2 in a dose-dependent manner; normally this receptor, when stimulated by CCL2 (MCP-1), induces monocyte migration and inhibits lymphocyte homing by modulation of CCL21-triggered integrin-mediated adhesions [30,31]. KOAs have also demonstrated the capacity to reduce edema formation, which is a common manifestation of inflammation. Further, in a rat model of Carrageenan-induced hind paw edema, CR845, Salvinorin A (Figure 1I), and U50-488H significantly reduced the hind paw volumes and the response to noxious stimuli [12,32]. Thus, the ability of KOAs to attenuate edema formation, inflammation, and inflammatory pain provides a unique opportunity for the development of a novel anti-inflammatory agent utilizing the kappa-opioid receptor pathways.

### 2.5. HIV-Induced Neuroinflammation

CXCR4 is a chemokine receptor responsible for promoting chemotaxis of lymphocytes and is expressed in all major CNS cell types, including neurons, astroglia, microglia, and oligodendrocytes [33]. The CXCR4 receptor has the capacity to regulate several signaling pathways, altering a variety of biological responses. In particular, CXCR4 signaling is vital to the pathological process of HIV infection. HIV can infect peripheral immune cells and use them to enter the CNS, where a cell reservoir is established and the CNS-immune cells promote inflammation. In doing so, the HIV-envelope protein from X4 viruses uses CXCR4 for entry into cells. Additionally, abnormal stimulation of CXCR4 can cause secretion of inflammatory mediators, thus promoting inflammation, whereas stimulation of CXCR4 by its natural ligand, CXCL12, is neuroprotective against virally-induced inflammation. Interestingly, individuals with HIV who use MOA analgesics tend to exhibit an increase in disease progression, thus outlining the potential interaction between the opioid and chemokine system in the CNS. Administration of selective MOAs inhibit the neuroprotective effects of CXCL12 treatment. However, treatment with the KOA U50,488H demonstrated the ability to desensitize CXCR4 signaling after acute administration and also to decrease CXCR4 surface expression during long-term administration. Additionally, administration of U50,488H decreased the transcription of CXCR4 by acting on the JAK/STAT pathway, leading to a decrease in X4 HIV infection. Thus, it has been proposed that KOAs may exhibit anti-inflammatory properties in the CNS in patients with HIV, whereas MOAs promote inflammation [34].

### 2.6. Anti-Emetic

Chronic nausea and vomiting decrease the quality of life and causing fatigue and irritability that can negatively impact mood and social interactions [35]. Additionally, nausea and vomiting are common side-effects associated with several medications, which is a significant predisposing factor to non-compliance. Commonly used anti-emetic medications, such as ondansetron (Zofran^®^), are effective in combating nausea and vomiting in most patients. However, for some patients, medication is either ineffective or contraindicated. Further, those taking serotonin-based drugs, such as tri-cyclic anti-depressants and selective serotonin re-uptake inhibitors (SSRIs), or for those with certain heart conditions, such as prolonged QT-syndrome, should avoid such medications due to the risk of potentially fatal side-effects. An alternative could be KOAs. It has been demonstrated that the DOR and peripheral MOAs promotes emesis, whereas KORs and central MORs have anti-emetic effects [36,37]. KORs have been detected in the chemoreceptor trigger zone within the area postrema in the floor of the fourth ventricle [38]. Thus, peripherally-selective KOAs may serve as viable candidates for the treatment of chronic nausea and vomiting.

### 2.7. Spinal Anesthesia

Spinal anesthesia is commonly used during lower abdominal, perineal, and lower extremity surgeries [39]. Anesthetic agents are administered into the subarachnoid space, allowing them to act directly on the spinal cord. Lidocaine is commonly used for lower body procedures due to its rapid induction and brief recovery period; however, lidocaine commonly promotes adverse neurological symptoms [40]. Opioids, such as fentanyl and sufentanil, are commonly co-administered with local anesthetics in order to promote the rapid onset and recovery from spinal anesthesia. Additionally, neuraxial administered MOAs can prolong intra- and post-operative analgesia [41]. However, neuraxial administration of such compounds commonly induce pruritus, which is very discomforting to the patient. Specifically, 83% of patients who receive neuraxial-opioids for a Cesarean section and 69% of patients for all other procedures experience pruritus. However, as stated previously, the KOR suppresses itch. It has been demonstrated that co-administration of U50,488-H with neuraxial-opioids attenuates the development of pruritus after spinal anesthesia [42]. Researchers in the Department of Anesthesiology at Shimane University of Japan demonstrated the ability of systemically administered TRK-820 to inhibit neuraxial-induced pruritus in primates. Furthermore, agonism of human spinal cord KORs are thought to promote analgesia [43,44]. A benefit of KOAs in spinal anesthesia is their ability to achieve significant analgesia without promoting respiratory depression. In a study conducted by faculty of the Department of Anesthesiology at the University of Alabama at Birmingham, researchers surgically implanted lumbar or cerebral ventricular catheters in Sprague–Dawley rats [45]. Subsequently, rats were administered various doses of U-50,488H and subjected to nociceptive testing. Results indicated that U-50,488H attenuates pain when administered directly into the spinal column or as an epidural, without promoting respiratory depression. Additionally, researchers at Hokkaido University of Japan found that the KOA U-50,488H antagonizes the respiratory effects of MOAs in rats when co-administered [46]. Thus, it is apparent that KOAs are a viable drug candidate for induction of spinal anesthesia and may serve as a valuable therapeutic to supplement the analgesic properties of MOA spinal anesthetics, while also attenuating the development of resultant pruritus.

### 2.8. Stroke and Neuroprotection

Stroke is the leading cause of long-term disability and the fifth leading cause of death in the United States [47]. Medical management of an ischemic stroke includes the intravenous administration of tissue plasminogen activator (tPA), also known as Alteplase [48]. Alteplase acts by dissolving the clot and restoring blood flow to the affected area of the brain; however, this process can lead to subsequent complications, including a hemorrhagic stroke and cerebral ischemia-reperfusion injury. Additionally, patients run the risk of developing cerebral edema as a result of ischemic stroke, which can lead to life-threatening complications such as brain herniation. KOAs have demonstrated the ability to mitigate the onset of both cerebral ischemia-reperfusion injury and cerebral edema. A study performed at Johns Hopkins University demonstrated that the highly selective KOA BRL52537 promotes neuroprotection by reducing ischemia-induced nitric oxide (NO) production in rats [49]. Subsequently, BRL52537 reduced cortical infarct size in rats following induction of global ischemia. In a study performed by Silvia et al., the KOAs U-50,488E and U-50,488H prevented brain edema and neuronal injury following transient global ischemia. This effect was thought to be due to the ability of KOAs to modulate glutamate- and NO-induced excitotoxicity [50]. In a rat model of transient focal ischemia from middle cerebral artery occlusion (MCAO), BRL52537 demonstrated a therapeutic window of six hours for neuroprotection [51,52]. Thus, stroke treatment and neuroprotection are promising areas for drug research and development of centrally-acting KOAs.

### 2.9. Hypoxic Pulmonary Hypertension

Hypoxic Pulmonary Hypertension (HPH) is seen as a result of various cardiac and pulmonary pathologies [53]. The unifying vasoconstriction and blood vessel remodeling causes increased pulmonary vascular resistance and resultant right-sided heart failure. Without transplant, the end result is often death. However, therapeutic intervention is limited by an incomplete understanding of the disease. It is known that chronic hypoxic stress promotes endothelial dysfunction and triggers remodeling of the pulmonary vasculature by promoting proliferation of pulmonary smooth muscle cells (PAMSCs). Also, the KOR is found on vascular tissue. A study conducted by Zhou et al. assessed the role of U50,488H and the quaternary ammonium salt form of U50,488H (Q-U50,488H), which has enhanced peripheral KOR selectivity, in attenuating HPH in rats, and the underlying mechanisms involved. Results indicated that Q-U50,488H suppressed pulmonary blood vessel remodeling under hypoxic conditions, which was attributed to decreased PASMC proliferation. In addition, both mean pulmonary arterial pressure (mPAP) and right ventricular pressure (RVP) were reduced in treated HPH rats. Interestingly, U50,488H had a slightly greater effect than Q-U50,488H in alleviating HPH. It was demonstrated that U50,488H was more efficient in promoting vascular relaxation than its quaternary salt counterpart. However, Q-U50,488H increased NO content by conserving endothelial nitric oxide synthase (eNOS) activity and by limiting NO destruction by elevating the total anti-oxidant binding capacity (T-AOC) and reducing the gp91^phox^ expression in the pulmonary artery. Lastly, Q-U50,488H led to the relaxation of the pulmonary artery rings. As anticipated, Q-U50,488H was attenuated by nor-BNI administration, thus supporting that the effects of Q-U50,488H are due to agonism of the KOR. Given the limited side-effect profile associated with peripherally-restricted kappa-opioid agonists, compounds such as Q-U50,488H could serve as a therapeutic for the treatment of HPH.

### 2.10. Multiple Sclerosis

Multiple sclerosis (MS) is a chronic demyelinating disorder of the CNS, affecting the brain, spinal cord, and optic nerves [54]. MS affects approximately 400,000 people in the United States and 2.3 million people worldwide, most of which are women. It is believed that the autoimmune destruction of myelin, which aids in neuroprotection and signal transduction, is triggered by an environmental exposure in genetically predisposed individual [54,55]. Myelination of the peripheral nervous system is mediated by Schwann cells, which demonstrate the capacity to remyelinate upon injury. However, myelination of the CNS is mediated by oligodendrocytes, which have limited capacity to promote remyelination. Currently, treatment of MS is centered on immunosuppression with agents such as corticosteroids, targeted monoclonal antibodies, and *β*-interferons; however, these agents are associated with debilitating and often life-threatening side-effects. Thus, a therapy that targets remyelination of CNS neurons with a limited side-effect profile would constitute a novel therapeutic treatment of multiple sclerosis, as well as other central demyelinating disorders. A study performed by the University of California, San Francisco demonstrated that KOAs promote oligodendrocyte precursor cell (OPC) differentiation into mature oligodendrocytes, with subsequent myelination [56]. Further, Mei et al. used rat oligodendrocyte cultures and human-induced pluripotent stem cell-derived OPCs to assess the effects of various KOAs in promoting myelination and oligodendrocyte differentiation. Lead compound U-50,488H emerged as the most efficacious of all compounds studied. In an in vivo model of focal demyelination with lysolecithin, U-50,488H promoted remyelination of CNS neurons. The effects of U-50,488H were abolished by administration of nor-BNI in all studies. The exact mechanism regarding this process is still unknown and thus, follow-up studies to reproduce these findings in greater detail would be of significant value. KOAs may be combined with corticosteroids during an acute demyelinating event to supplement the immunosuppressive effects of drugs such as prednisone, while also promoting remyelination. However, as previously stated, centrally-acting KOAs are associated with several untoward side-effects. Thus, the development of pathway-specific KOAs might offer a regenerative option for treating central demyelinating disorders.

### 2.11. Addiction

Approximately 20.6 million people in the United States suffer from illicit drug addiction, with over 3 million people actively seeking treatment per year. Addiction is a complex disease that affects brain function and behavior [57]. There are a variety of treatment options available for addiction; however, no single treatment is right for everyone. While many patients respond well to psychotherapy and group rehabilitation programs, others respond better to pharmacotherapy. It has been demonstrated that addictive drugs increase extracellular levels of dopamine in the nucleus accumbens [58]. Further, KORs neighbor dopamine transporters (DAT) in neurons expressing tyrosine hydroxylase activity. In the nucleus accumbens, KOA administration has been shown to decrease extracellular dopamine (DA) concentrations through the inhibition of DA release, as well as through upregulating DAT reuptake activity [59,60].

In a study performed at Albany Medical College, Maisonneuve et al. demonstrated that the KOA U-50,48H attenuates cocaine-induced increases in extracellular DA in the nucleus accumbens of rats [58]. A study performed by Victoria University of Wellington, New Zealand demonstrated that the administration Salvinorin A also modulated extracellular dopamine levels and attenuated cocaine-prime-induced reinstatement in rats [61]. Additionally, KOA U-69,593 (Figure 1J) decreased cocaine self-administration in rhesus monkeys. For these reasons, novel KOAs may one day serve to dampen the significant morbidity and mortality associated with drug addiction.

### 2.12. Post-Traumatic Cartilage Regeneration

Osteoarthritis (OA) is the most common form of arthritis, with over 3 million cases reported in the United States every year. While any joint can be affected, the disorder is most commonly observed in the hands, knees, hips, and spine [62]. Treatment of OA consists of reducing pain and preserving range of motion. Pain management frequently includes NSAIDs, and, less commonly, MOAs for severe cases. However, no therapy currently available directly targets damaged cartilaginous tissue. Recently, KOR expression was found on chondrocytes and synovial cells. Preliminary efforts have revealed multiple routes of cartilage regeneration after KOR activation; however, further delineation of these exact mechanisms is needed [63]. Additionally, Wu et al. demonstrated that the KOR is highly expressed during human development in joint-forming cells.

In a model of osteoarthritis, KOR knock-out mice had accelerated cartilage degeneration following injury when compared to wild-type mice. KOR activation increased anabolic enzyme expression and reduced cartilage breakdown in response to TNF-α. Also, selective KOR agonism on chondrocytes and synovial cells stimulated downstream cAMP/CREB pathways, which ultimately promoted better joint lubrication. These results highlight the protective effects of KOR signaling in joint tissue after injury. Thus, KOAs may offer a combined benefit of joint protection and pain control in patients with newly diagnosed osteoarthritis. Studies assessing the effects of the KOA JT09 on mitigating post-traumatic cartilage degeneration are currently ongoing at the University of Southern California.

## 3. Conclusions

KOAs are as efficacious as MOAs in treating acute and chronic pain; however, the development of KOAs has been limited due to untoward CNS-mediated side-effects, such as dysphoria, sedation, hallucinations, and psychosis. Thus, there have been recent efforts to develop pathway-biased KOAs that act on beneficial signaling pathways, as well as efforts to develop peripherally-restricted KOAs that have limited CNS penetration. Both approaches reduce the risk of adverse effects associated with conventional KOAs. As previously stated, KOAs have been well described as prospective non-addictive alternatives for the treatment of pain. Further, the MORs and DORs promote euphoria, respiratory depression, and physical dependence [64]. In contrast, opioids targeting central KORs cause little to no respiratory depression, and produce dysphoric effects rather than euphoria, which reduces their dependence potential [64]. Additionally, spinal and peripheral MORs reduce intra-gastric pressure and phasic contractions of the gastrointestinal tract, thus leading to constipation [65]. KOAs do not weaken intestinal mobility or foster constipation, thus KOAs offer advantages over MOAs and are being studied as an alternative therapeutic for pain management. Previously recognized KOA activity includes anti-nociceptive, cardiovascular, anti-pruritic, anti-edematous, diuretic, anti-inflammatory, and anti-tussive effects. In addition, KOAs have demonstrated efficacy in models of stroke, spinal anesthesia, hypoxic pulmonary hypertension (HPH), multiple sclerosis, addiction, and post-traumatic cartilage regeneration.

Despite the diverse and potent activity of KOAs in a variety of disease indications, there are still many unanswered questions and potential barriers to the development of such compounds. Furthermore, KOAs act as aquaretic agents, promoting diuresis. While diuretic activity is desired in patients with cardiovascular disease, it may lead to serum electrolyte abnormalities, such as hypernatremia [12]. The KOA CR845 was placed on a phase II clinical hold due to the promotion of hypernatremia at the highest intravenous dose administered. Of note, KOAs act similar to *β*-blockers by exhibiting negative chronotropic and inotropic effects on the heart. Moreover, KOAs play a role in autonomic nerve cross-talk by inhibiting neurotransmitter release and cardiac responsiveness to hormones such as norepinephrine [66]. Thus, KOAs may potentiate the side-effects of *β*-blockers when co-administered together. Additionally, the cardiac depressant effects induced by KOR stimulation may potentially exacerbate the diminished cardiac function observed in end-stage heart failure, limiting KOA administration to early-stage heart failure [67]. Lastly, KOAs have potent anti-inflammatory and immunosuppressive effects. While desirable in certain conditions, such effects may lead to untoward side-effects, including increased risk of infection, delayed wound healing, and malignancy, with long term administration. Notwithstanding potential barriers, the diverse activity of KOAs warrants further development as a drug class [68].

In conclusion, pathway specific or peripherally-restricted KOAs present an excellent opportunity to develop novel therapeutic agents that have a limited side-effect profile, with efficacy in a wide-range of indications. Thus, KOAs have the potential to prevent, improve, and treat a wide-range of health problems.
